# An innovative data mining-driven optimisation modelling approach based on TAM for the design of elderly-centric ICT products

**DOI:** 10.1038/s41598-026-37657-x

**Published:** 2026-01-24

**Authors:** Yu Cao, Xiaogang Yang, Shijian Luo, Kok-Hoong Wong

**Affiliations:** 1 Faculty of Science and Engineering, University of Nottingham Ningbo China, Ningbo, 315000 China; 2 Department of Industrial Design, College of Computer Science and Technology, Zhejiang University, Hangzhou, 310027 China

**Keywords:** Design and manufacturing, Design optimisation model, Elderly-centric products, Technology acceptance model, Data mining, Latent dirichlet allocation, Engineering, Mathematics and computing

## Abstract

To enhance the quality of life and health of the elderly, it is necessary to incorporate their specific needs into the design and manufacturing processes of Information and Communication Technology (ICT) products. However, these needs are often overlooked in product development, leading to poor user experiences and low adoption rates. To address this important issue, this study innovatively proposes a data mining-driven optimisation modelling approach. This approach first extracts key design elements from online reviews of elderly ICT products through text mining techniques such as data purification and Latent Dirichlet Allocation (LDA). These elements are then incorporated as external variables to construct a user needs integrated technology acceptance model (UN-TAM) for the elderly, which is validated using Structural Equation Modelling (SEM). Using the elderly smartwatch as a case study, four design optimisation parameters, functional architecture, human-computer interface, morphological aesthetics, and interaction mode, were extracted from a substantial database based on the elderly user comments. The proposed model establishes a quantitative mapping relationship among “design parameters, user perception, and behavioral intention”. The results indicate that these design parameters significantly influence the product acceptance through three distinct pathways: perceived ease of use (γ = 0.641, *p* < 0.001), perceived usefulness (γ = 0.601, *p* < 0.001), and perceived enjoyment (γ = 0.402, *p* < 0.001). Specifically, functional architecture of the elderly-centric ICT products has the most substantial impact on perceived usefulness while the other three parameters predominantly influence perceived ease of use. The proposed data mining-driven optimisation modelling approach can be effectively used in designing and manufacturing elderly-centric ICT products.

**Supplementary Information:**

The online version contains supplementary material available at 10.1038/s41598-026-37657-x.

## Introduction

Aging has emerged as a significant global issue. The United Nations Department of Economic and Social Affairs projects that the population aged over 65 will rise from 530 million to 1.5 billion by 2050, increasing from 7.7% to 15.6% of the total population^1^. This trend indicates a growing demand for health care, assistive living, and independent living solutions for the elderly^2^. Concurrently, Information and Communication Technology (ICT) has advanced rapidly and become integral to daily life, offering potential to meet the physiological and psychological needs of the elderly in an era marked by both aging and digitisation. ICT encompasses digital applications and devices that enhance access to information and facilitate communication through online platforms. Extensive research highlights ICT’s benefits in improving elderly people’s well-being^3^, social connectedness^4^, and healthcare^5^. It is believed that ICT can bridge the gap between the desires and needs of the elderly, enhance their quality of life, and reduce social care costs^6^.

While ICT exhibit immense potential in promoting healthy ageing, the engagement of the elderly demographic with ICT applications remains markedly insufficient^7^. Research indicates that, compared to other age groups, the elderly encounter multiple barriers when utilising ICT products: limitations in digital literacy, a lack of technological knowledge, and cognitive and physical declines associated with aging. These factors collectively contribute to the exclusion of this demographic from ICT technologies^8^. A more in-depth analysis reveals that, due to significantly lower familiarity with technology than younger generations, older individuals commonly experience technological anxiety and a lack of confidence in usage^9^. Such psychological impediments further diminish their likelihood of adopting and effectively using ICT products^10,11^. These findings illuminate a critical issue: the current design paradigms of ICT products fail to adequately address the unique needs of elderly users, transforming what should be conveniences into new obstacles. Consequently, it is imperative to improve ICT product design based on the physiological characteristics, cognitive traits, and usage habits of the elderly^12^; this not only represents a necessary requirement for enhancing technological inclusivity but also serves as a vital pathway towards achieving the strategic goals of healthy ageing.

The technology acceptance model (TAM) proposed by Fred D. Davis in 1989 is an effective model in the field of user behavior research^13^. In existing model applications, researchers typically select different external variables based on various subjects of study to examine the impact of these variables on user acceptance of new technologies or products, and based on the results of the studies, new technologies or products are adapted to fulfil users’ needs^14,15^. In recent years, an increasing number of scholars have focused on the lack of acceptance of ICT products by the elderly and have used TAM as a theoretical basis to assess and predict whether the elderly are able to successfully access information technology^16,17^. These studies confirm the explanatory power of TAM for the elderly’s acceptance of ICT products.

Nevertheless, there are still two deficiencies in using TAM to investigate the acceptance of ICT among elderly users. First, the conventional TAM development process predominantly relies on literature-derived external variables^18–21^, while neglecting direct input from elderly users themselves. This approach may overlook factors that elderly people are genuinely concerned about, due to the discrepancy between researchers’ perspectives and those of elderly users^22^. Currently, only a few studies involve elderly users in TAM research by conducting interviews and coding with them^23,24^. This lack of engagement stands in stark contrast to the fundamental principles of age-friendly design. As highlighted by G. Rodeschini et al.^25^, designers should view the elderly as active participants rather than merely recipients of technology when creating designs for this demographic. Dodd et al.^26^ also argue that senior users have drastically different needs than younger users, and solutions that address these needs require some sort of participative or human-centered design. Thus, in order to enhance the explanatory power of TAM regarding the adoption of ICT by the elderly, and to provide a foundation for subsequent design optimisation, it is essential to systematically incorporate the perspectives of the elderly during the model’s development stage.

Second, technological or product factors are often treated as a single entity in the most studies. In the application of TAM, discussions at the technical level tend to focus on users’ perceptions of the technology^21,23^, or consider technology-related factors as a whole^27,28^. This macro-level analysis makes it difficult for research to reveal the mechanisms by which specific design elements interact with the internal perceptions of the elderly. Although some scholars have acknowledged this issue^11,27^, existing research remains deficient in the micro-deconstruction of design elements, failing to provide concrete theoretical guidance for the age-friendly design. We also note that, distinct personal characteristics, such as health status^18,19^, education level^28^, social influence^19^, and facilitating conditions^21^ are frequently chosen as the external variables. However, the individual characteristics of the elderly users are fixed. Exploring the differences in acceptance levels of ICT caused by individual differentiated factors is of limited significance in enhancing the inclusivity and age-friendliness of technologies or products. This approach resembles a process of selecting users, which contradicts the principles of user-centered design. Therefore, based on the research gaps mentioned before, the main research aims of this study is:

1. Developing a user-needs integrated technology acceptance model (UN-TAM) for the elderly through a data-driven approach.
2. Incorporating product design elements into TAM to investigate their influence on the acceptance of ICT products by the elderly.

**Fig. 1:**
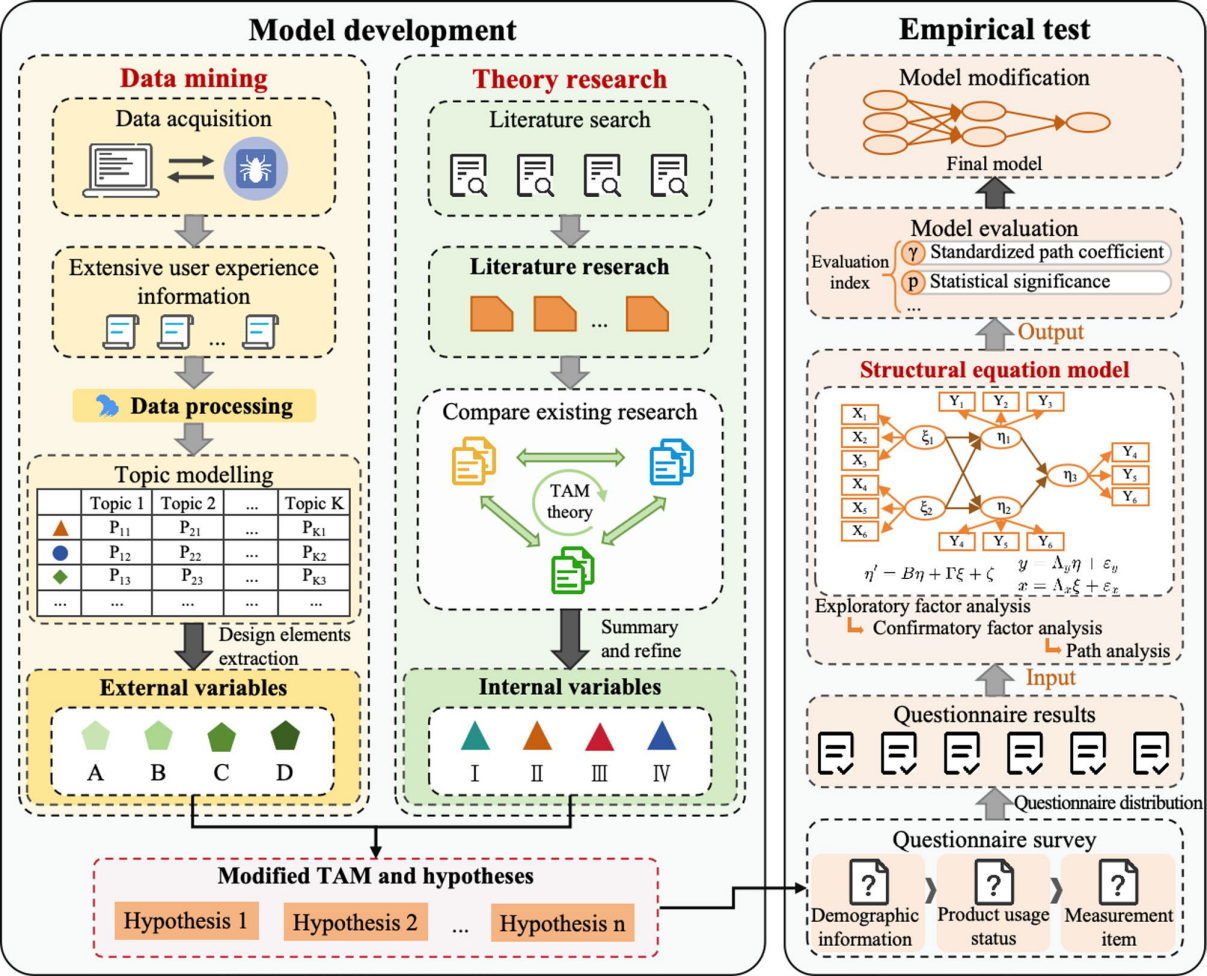
The research approach and process.

User needs can be derived from mining elderly user comments online. In practice, an innovative screening mechanism for elderly users’ comments is tested and validated, which combine data mining methods to capture elderly users’ needs from massive online comments. The latent Dirichlet allocation (LDA) topic modelling is then used for topic modelling to identify the core design requirements. The paper is organized as follows. “Model development and hypotheses” introduces the model development path and hypotheses. “Results” and “Discussion” give the research results and the related discussion, respectively. In “Conclusions”, some concluding remarks are presented. The research approach and process are illustrated in Fig. 1.

## Model development and hypotheses

### Data mining approach for elderly user needs acquisition

Data mining technique is employed to collect and analyze comments from the available online shopping platforms, focusing on the needs of elderly users. Data mining, especially text mining, is a technique for extracting the valuable information from the large amounts of text data. It involves multiple fields such as natural language processing, machine learning, and statistical analysis^29^. In the field of user needs research, text mining provides an efficient method for analyzing and interpreting the vast amounts of user-generated text data, thereby revealing users’ true needs and preferences^30^.

**Fig. 2:**
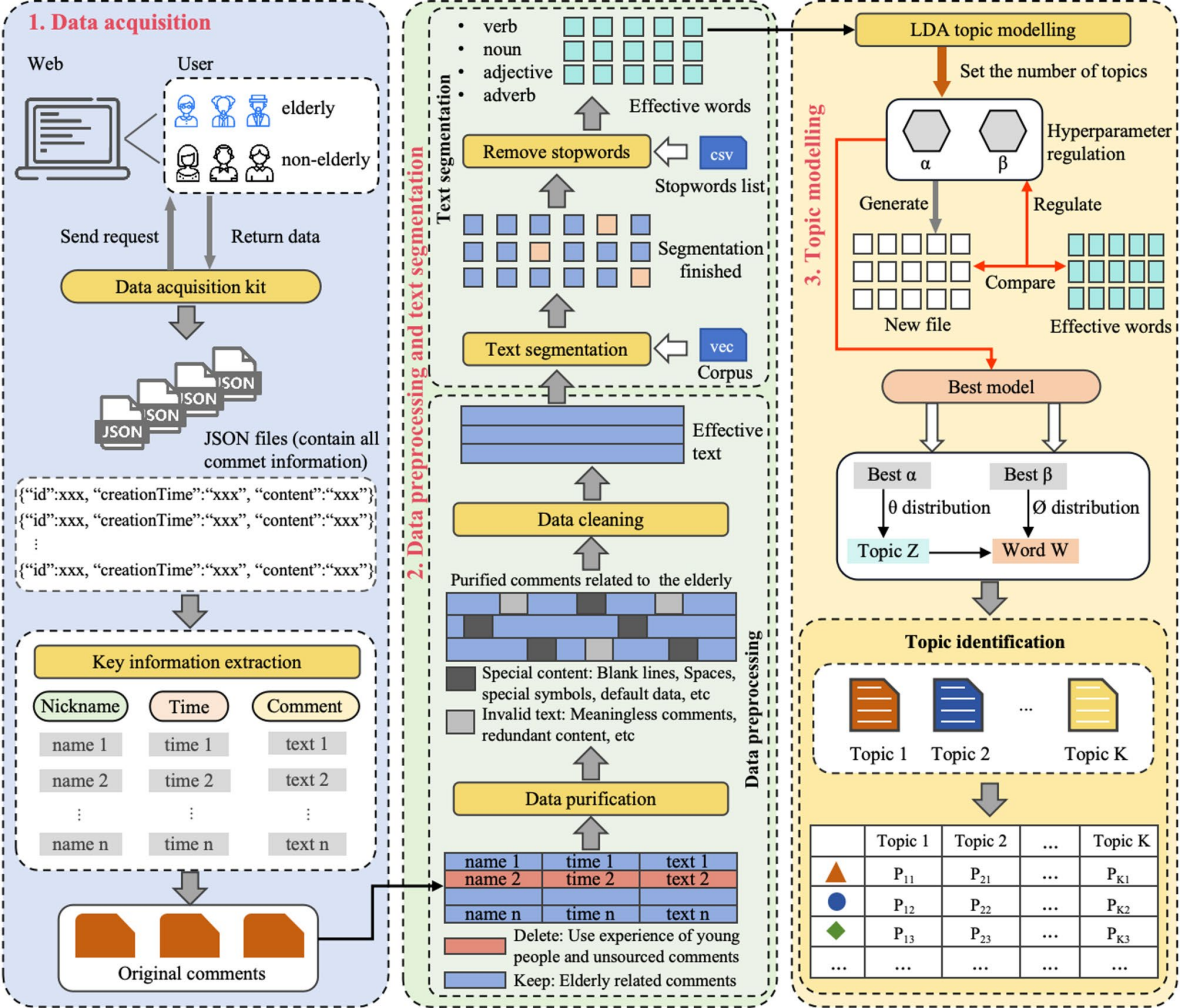
The consumer groups of elderly smart products, as well as the principle and technical path of elderly user needs acquisition based on data mining.

The process of acquiring elderly user needs based on the data mining includes the steps such as selection of the online shopping platforms and products, data collection, data preprocessing, text segmentation, and topic modelling. LDA is used to perform the topic modelling and classification on those diverse reviews to identify the core needs. LDA is an unsupervised machine learning algorithm with the goal of estimating model parameters through an iterative algorithm to discover the thematic structure in a collection of documents^31,32^. This study will utilize the LDA method to identify latent topics in the text data of elderly user reviews, with the aim of recognizing and understanding their core needs. Figure 2 illustrates the consumer groups of elderly ICT products and the technical paths for acquiring elderly user demands based on the data mining adopted in this study.

In this study, “JD.COM”, one of the largest domestic e-commerce platforms in China, was chosen as the source for data collection. The elderly smartwatch was selected as a case study as one of the representative ICT products which are relatively accessible and available. Python web scraping was utilized to extract comments from product pages. The JSON data returned by the web pages was then parsed into a Python-readable data structure. As of January 16, 2023, a total of 179,824 user comments were scraped from the product pages of 33 elderly smart watches available on JD.COM.

The data mining methodology employed in this study derives its distinctiveness and innovation from the introduction of a crucial “data purification” pre-processing step. Due to the inherent openness of online platforms, individuals of all ages can access any variety of products. Consequently, consumers and users of elderly-targeted ICT products are not exclusively senior citizens, which implies that not all user reviews stem from the feedback of elderly users—a fact evidenced by the intricate and diverse nature of online commentary. Thus, we constructed a keyword repository pertaining to the elderly and manually filtered comments explicitly associated with elderly users. This step ensures the purity and representativeness of the subsequent analytical data source, forming the fundamental prerequisite for the accuracy and reliability of the subsequent topic modelling results. Specifically, three main consumer groups can be identified through the observation and analysis of these online comments. The first group consists of elderly individuals who purchase products for themselves or their partners. They typically use keywords such as “old guy”, “old lady”, “old companion”, “advanced in age”, “getting on in years” and “elderly”, which reflect their perspective as elderly individuals. The second group comprises young people who purchase products for their parents or elders. They indirectly express the feelings of the elderly towards the products, using keywords like “dad”, “mom”, “elderly person”, “senior”, “grandma”, “grandpa”, “auntie”, and “mother-in-law”. Lastly, there is a group of individuals whose age is unknown, making it challenging to determine whether they are elderly, and this group constitutes most of the comments (more than 50%). Therefore, only comments from the first two categories that clearly indicate the experiencers and evaluators as elderly individuals are selected to ensure data purity. Keywords related to the elderly were identified through analyzing various consumer reviews, mainly including terms for addressing elderly individuals and descriptions of older age. A manual search and selection process was conducted to filter 8840 review contents related to elderly users based on these keywords. Some original review data related to the elderly is shown in Table 1.

**Table 1:** Some typical and original review data related to the elderly.

Index	ID	Comment time	Comment content
1	****2	2023/1/3 19:16	It is very convenient for the elderly, and you can understand the elderly’s heartbeat, blood pressure, blood oxygen saturation and body temperature in real time. The operation is simple, and the delivery speed is fast
2	****j	2023/1/1 20:29	Sensitivity: very spirit; Accuracy: very good; Operation difficulty: the elderly can also operate; Quality of workmanship: in good workmanship; Appearance: stable appearance
3	****S	2022/3/13 15:50	I chose this one for my family. It is very convenient to use, and my father has consistently had high blood pressure
4	****w	2023/1/5 10:13	The watch is received, consistent with the description, and easy to operate, very suitable for the elderly to wear
…	…	…	…

Data cleaning was followed, including special content processing and invalid text processing. After manually filtering to obtain valid textual data, JIEBA, a commonly used open-source word segmentation tool in the Python community, was employed for text segmentation. Furthermore, enhance the accuracy and efficacy of text analysis by removing the stop words such as punctuation, special characters, emoticons, and discourse markers. After text segmentation and removal of stop words, a total of 470 meaningful words were retained, with frequencies ranging from 1 to 536.

Subsequently, LDA model within the Python environment was utilized to conduct topic modelling on the tokenized results, thereby generating distinct topics that encapsulate the various aspects of product design. The LDA model class from the Genism library, specifically version 4.2.0, was employed to implement the LDA. It should be noted that the value of num_topics is determined as 10 by the topic coherence score. Suppose the top-M keyword set for a topic is $\:\left\{{w}_{1},{w}_{2},\cdots\:,{w}_{M}\right\}$, then the formula for calculating the topic coherence score $\:{C}_{v}\left(model\right)$ is as follows (Eqs. 1–3):

1 $$\:{C}_{v}\left(model\right)=\frac{1}{K}\sum\:_{k=1}^{K}{C}_{v}\left({topic}_{k}\right)$$

2 $$\:{C}_{v}\left(topic\right)=\frac{2}{M(M-1)}\sum\:_{i=1}^{M-1}\sum\:_{j=i+1}^{M}sim({w}_{i},{w}_{j})$$

3 $$\:sim\left({w}_{i},{w}_{j}\right)=\mathrm{cos}\left({\overrightarrow{v}}_{{w}_{i}},{\overrightarrow{v}}_{{w}_{j}}\right)=\frac{{\overrightarrow{v}}_{{w}_{i}}\cdot \:{\overrightarrow{v}}_{{w}_{j}}}{||{\overrightarrow{v}}_{{w}_{i}}||\cdot \:||{\overrightarrow{v}}_{{w}_{j}}||}$$

In the formula, K denotes the total number of topics in the model; M denotes the number of top keywords within a topic; $\:{\overrightarrow{v}}_{{w}_{i}}$ and $\:{\overrightarrow{v}}_{{w}_{j}}$ represent the context vectors of words $\:{w}_{i}$ and word $\:{w}_{j}$ respectively, $\:{\overrightarrow{v}}_{{w}_{i}}\cdot \:{\overrightarrow{v}}_{{w}_{j}}$ denotes the vector dot product, and $\:|| \cdot ||$ denotes the vector norm.

**Fig. 3:**
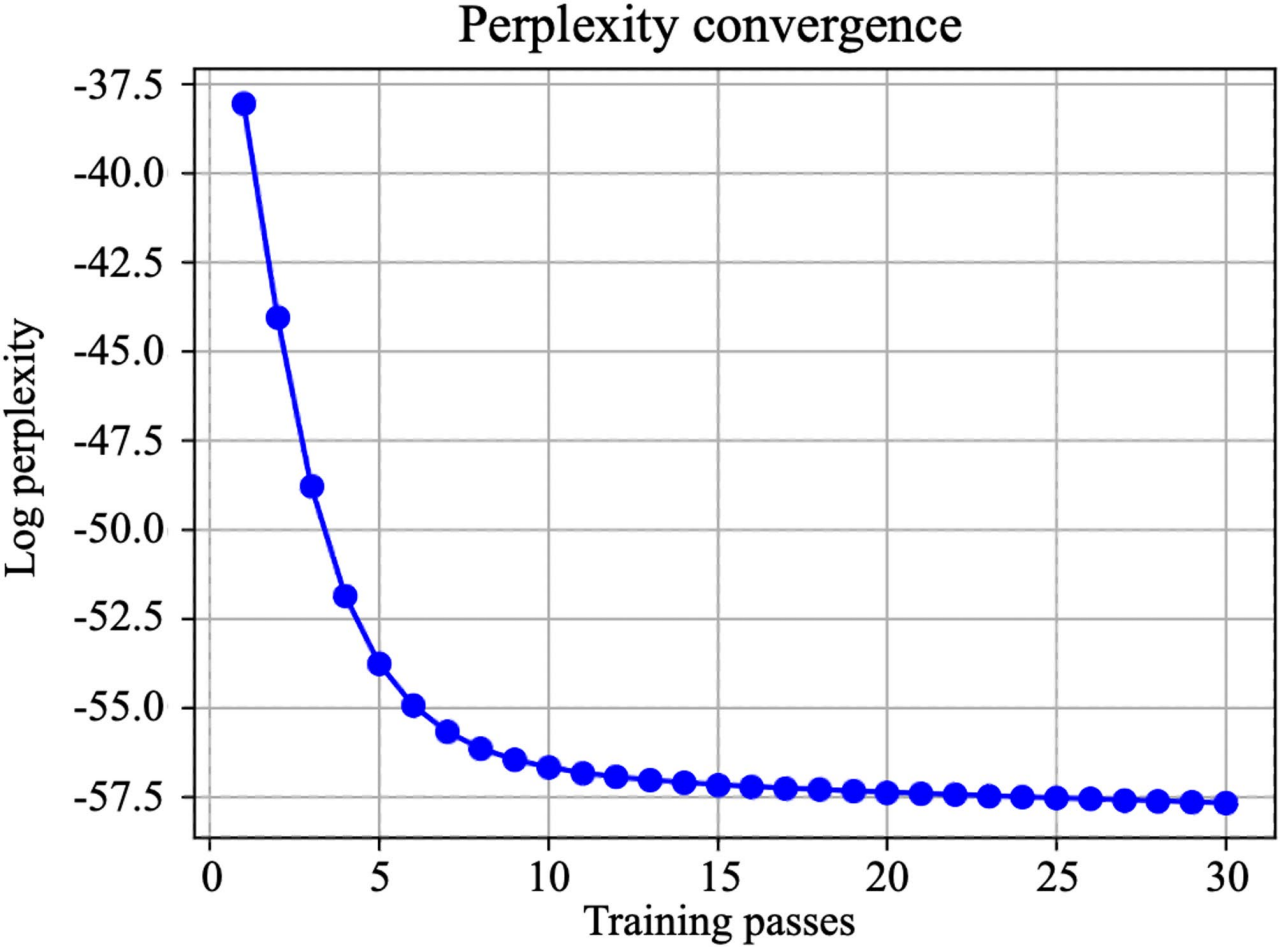
Log perplexity convergence curve for the LDA model.

**Fig. 4:**
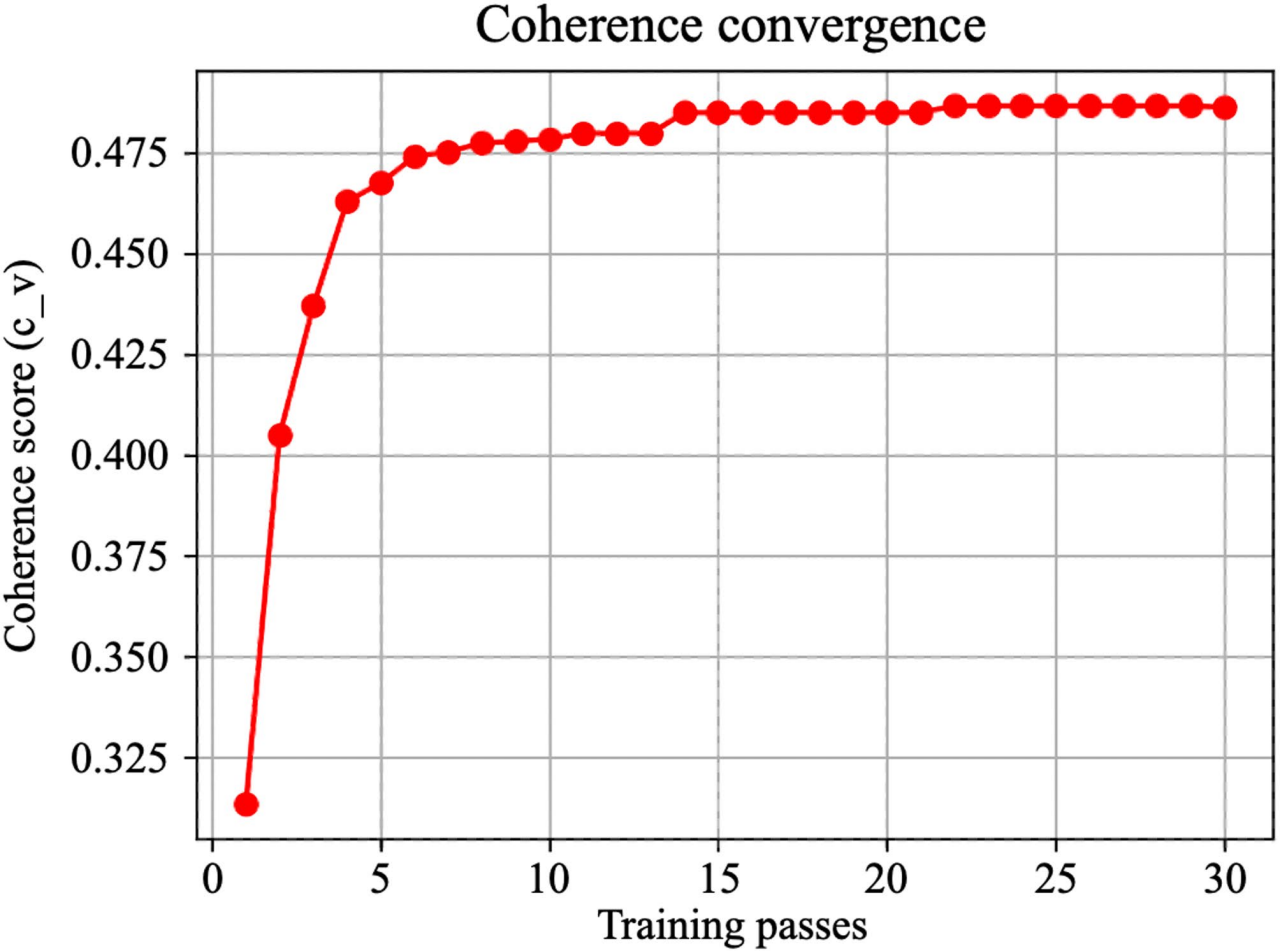
Coherence score convergence curve for the LDA model.

Preliminary experiments were conducted to determine the optimal value for passes. As evidenced by the log perplexity curve (Fig. 3) and coherence curve (Fig. 4), the model converged stably when passes = 30. Consequently, passes was set to 30. The hyperparameters of the established model are detailed in Table 2.

**Table 2:** The hyperparameters of the established model.

Hyperparameter	Description	Value
corpus	Corpus	corpora.Dictionary(Data)
num_topics	Number of topics that the model attempts to extract from the review segmentation results	10
id2word	Dictionary for mapping word ids to actual words	pos_dict.doc2bow(i)
passes	Number of iterations	30

To represent the core content of each topic, twelve words were selected from each topic. These were the words appearing most frequently within that topic. The weighting coefficient preceding each word indicates its frequency of occurrence within its respective topic. Ultimately, it was determined that ten topics, each comprising twelve words, yielded the most comprehensive results. Examples of the topic modelling outcomes are presented in Table 3.

**Table 3:** LDA topic modelling results.

Topic modelling results	
No.1	0.102×function + 0.072×positioning + 0.038×blood pressure + 0.035×measure + 0.021×volume + 0.020×comfortable + 0.019×heart rate + 0.014×appearance + 0.013×monitor + 0.013×shape + 0.013×call + 0.012×large
No.2	0.081×function + 0.042×positioning + 0.040×blood pressure + 0.036×monitor + 0.026×heart rate + 0.023×sound + 0.022×handy + 0.020×good-looking + 0.014×large + 0.014×signs + 0.012×assess + 0.011×voice
…	…
No.10	0.082×blood pressure + 0.066×function + 0.032×appearance + 0.031×measure + 0.031×quality + 0.026×sensitivity + 0.025×workmanship + 0.024×heart rate + 0.024×accuracy + 0.022×shape + 0.022×precision + 0.020×blood pressure

However, as LDA is an unsupervised algorithm, the connections among topics are ambiguous, resulting in some redundancy. After consulting with product design specialists and considering the relationships between the terms used in each topic, four main components of product design were determined to be “functional architecture,” “morphological aesthetics,” “interaction mode,” and “human-computer interface.” Simultaneously, the specific design requirements for various design elements were summarized by combining the segmentation results (see Table 4).

**Table 4:** Design elements and user needs of elderly smartwatches on online reviews.

Design elements	Part of speech	Related vocabulary	Frequency	Total frequency	User needs
Functional architecture	a. Adjective b. Noun	a. Convenient, accurate, practical, sensitive, powerful, complete b. Function, performance, blood pressure, position, location, blood pressure measurement, sphygmomanometer, electrocardiogram, blood oxygen, telephone, call, alarm, heart rate, monitoring, reminder, measurement, surveillance, detection, testing	a. 1373 b. 3519	4892	Vital signs monitoring function Positioning function Call function One-touch alarm function Monitoring accuracy
Interaction mode	a. Adjective b. Noun	a. simple, easy, difficult, complex, easy to learn, easy to understand, sensitive b. Key, button, touchscreen	a. 575 b. 66	641	Friendly interaction Smooth operation
Morphological aesthetics	a. Adjective b. Noun	a. Good-looking, beautiful, exquisite, generous, fashionable, compact, simple, aesthetically pleasing, delicate b. Appearance, shape, exterior design, appearance design, looks, color	a. 287 b. 348	635	Design Volume Color
Human-computer interface	a. Adjective b. Noun	a. Clear, distinct, concise b. Interface, screen, font, character	a. 130 b. 101	231	Appropriate size of screen and font, Reasonable interface layout

### Hypotheses development

To explore the impact of different product design elements on user acceptance and usage of digital products, a model and hypotheses are established based on TAM. Within the framework of TAM, perceived usefulness (PU) and perceived ease of use (PEOU) are consistently essential core variables, regardless of how TAM evolves^15,33^. They play a crucial role in directly influencing users’ attitudes and behavioral intentions towards usage, which explains why the modified TAM model, characterized by its concise structure and robust measurement, is the most widely used^15^. PU and PEOU are accepted as two main constructs affecting the behavioral intention (BI) to use ICT. That is, the more ICT is viewed as useful and easy to use, the more positive the influence on user acceptance. Furthermore, perceived enjoyment (PE) refers to “the degree to which using a technology is perceived as fun”^34^. Bargas-Avila and Hornbæk^35^ contend that hedonic qualities, including aesthetics, enjoyment, and the sense of identification experienced during interaction are critical. Therefore, perceived usefulness, perceived ease of use, perceived enjoyment, and behavioral intention are finally selected as the internal variables for the constructed TAM.

The model’s external variables are derived from data mining of online reviews, ensuring objectivity in assumptions and focusing on core design elements. Product function is the most common subject of comments in online reviews of elderly smartwatches. Typically, it serves as the most direct motivator for elderly users to purchase and use, as the elderly population typically exhibits utilitarian preferences. Moreover, diverse innovative functions are among the most significant differences between smart products and traditional ones. Therefore, the functional architecture (FA) of smart products inevitably plays a crucial role in shaping the perception and experience of elderly users. Morphological aesthetics (MA) serves as a crucial cue for users’ emotional intentions, significantly influencing their evaluations of the product. MA with innovation and aesthetical resonant is a vital means to enhance product market competitiveness. The abundant vocabulary used to assess MA in online reviews of elderly smartwatches reflects the elderly users’ pursuit of aesthetic experiences.

Interaction mode (IM) refers to the interface logic and interaction methods during the use of smart products, which can be categorized into physical interaction and interface interaction^36^. The diverse interaction modes pose a challenge for contemporary elderly users, compared to traditional products. Online reviews indicate that elderly users often express positive attitudes toward products characterized as “easy to use,” “easy to learn,” and “simple to operate.” Therefore, the complexity of IM is closely related to the acceptance level of elderly users towards the product. The human-computer interface (HCI) directly determines whether users can smoothly engage in deep interaction and use the product normally. The HCI of elderly smartwatches includes handling design elements such as screen size, fonts and icons, and interface layout. From online reviews, it can be observed that HCI is a commonly considered aspect among elderly users. HCI serves as the gateway for human-computer interaction, theoretically impacting the acceptance and usage of smart products by the elderly^37^.

Ultimately, functional architecture, morphological aesthetics, interaction mode, and human-computer interface are selected as the external variables of TAM to explore their impact on elderly user acceptance. Therefore, to explore the influence of different product design elements on elderly users’ perceptions, the following hypotheses are proposed based on the results of data mining:

H1: The functional architecture of smartwatches positively influences perceived ease of use (H1a), perceived enjoyment (H1b), and perceived usefulness (H1c) of elderly users;

H2: The morphological aesthetics of the smartwatches positively influences perceived ease of use (H2a), perceived enjoyment (H2b), and perceived usefulness (H2c) of elderly users;

H3: The interaction mode of the smartwatches positively influences perceived ease of use (H3a), perceived enjoyment (H3b), and perceived usefulness (H3c) of elderly users;

H4: The human-computer interface of the smartwatches positively influences perceived ease of use (H4a), perceived enjoyment (H4b), and perceived usefulness (H4c) of elderly users.

Elderly users are likely influenced by their emotional experience, which may affect their future intention to use ICT^3^. Existing research has shown that enjoyment from using a product can positively influence the intention of elderly individuals to use new products^38,39^. Ramírez-Correa et al.^38^ have confirmed that the PEOU of elderly individuals positively promotes PE in studying the use of hedonic information systems. Therefore, the following hypotheses are proposed:

H5-H6: Elderly users’ perceived ease of use (H5) and perceived usefulness (H6) of the smartwatch positively influences their perceived enjoyment.

H7: Elderly users’ perceived enjoyment of the smartwatch positively influences their behavioral intention towards it.

Perceived usefulness and perceived ease of use play a crucial role in directly influencing users’ attitudes and behavioral intentions. In the case of senior citizens, research supports that perceived ease of use and perceived usefulness predict the intention to use ICT^40^. Therefore, the following hypotheses are proposed:

H8-H9: Elderly users’ perceived ease of use (H8) and perceived usefulness (H9) of the smartwatch positively influences their behavioral intention towards it.

**Fig. 5:**
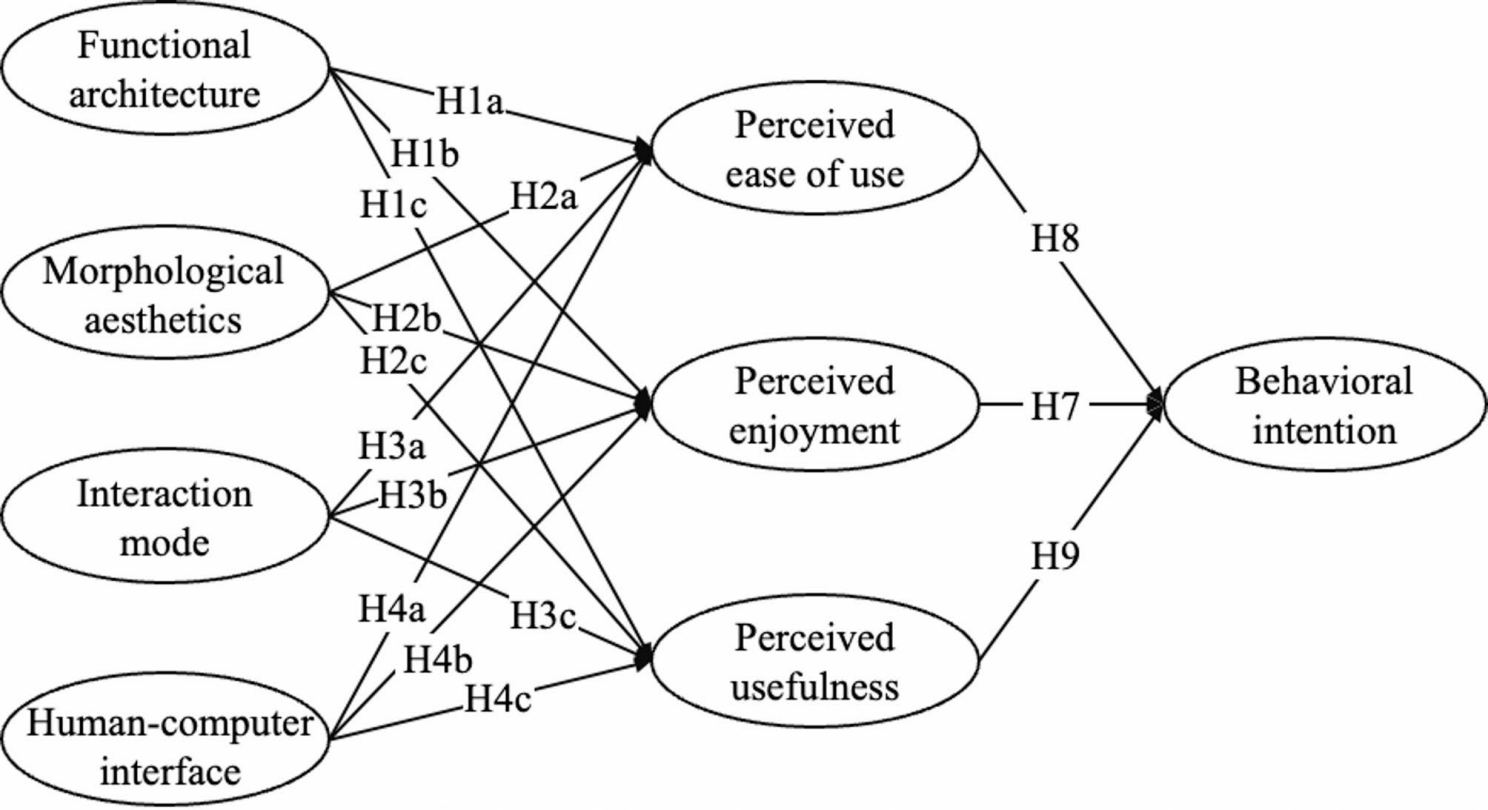
The original framework of UN-TAM based on the proposed hypotheses.

Based on the research of the previous stage, select relevant product design elements as the external variables of the UN-TAM (Fig. 5). The internal variables of the model are derived from the widely applied TAM modification models and the analysis of the physiological and psychological characteristics of elderly users. This facilitates the study on the influence of different product design elements on the internal perception of the elderly. The original framework of UN-TAM is shown in Fig. 3.

### Questionnaire development

A questionnaire was developed to empirically test the constructed model and hypotheses. The questionnaire consists of three parts. The first part involves gathering demographic information, such as age, gender, education level, and income level. The second part investigates the elderly population’s understanding and usage of smartwatch products. The content of the first two parts is intended to provide insights into the varying choices of users. The third part is the core of the questionnaire, in which elderly users rate the measurement items of various variables using a five-point Likert scale. Each variable is measured using 2 to 4 items. The measurement variables and item settings are based on Supplementary Table 1.

In order to achieve a larger sample size, questionnaires were distributed both online and offline, utilizing random sampling methods to survey individuals aged 60 and above. Previous research has indicated that online and offline (paper-based) questionnaires can be used interchangeably with similar effectiveness^41,42^.

We have confirmed that all experiments were conducted in accordance with relevant guidelines and regulations. According to Article 32 of the *Guidelines for Life Sciences and Medical Research Involving Humans* issued by the National Health Commission of the People’s Republic of China, ethical review is exempted when the research involves the use of human information or biological samples without causing harm to individuals, does not involve sensitive personal information, and is unrelated to commercial interests^43,44^. As our study meets these criteria, our study did not undergo formal ethical review. The questionnaire and methodology for this study were approved by the Human Research Ethics Committee of the University of Nottingham (Research Ethics Checklist: REQ2025080511). All participants were anonymous and participated voluntarily. Prior to participation, participants were informed of the purpose of the study, the relevant participation procedures, and their rights to participate, refuse participation, or withdraw at any time. They were also informed that their responses would be kept confidential and used solely for academic purposes. The collected data did not include any personally identifiable information, and informed consent had been obtained from all participants.

The survey began on February 17, 2023, and lasted for 18 days. Offline data collection primarily focused on parks and squares in Wuhou District, Chengdu, Sichuan Province, resulting in 32 questionnaires. Meanwhile, 150 online questionnaires were collected from respondents in Sichuan, Anhui, and Shandong provinces through social media like WeChat. As a result, 182 questionnaires were collected, of which 8 were considered invalid due to incompleteness or inconsistencies. Thus, 174 valid questionnaires remained, resulting in an effective rate of 95.6%.

###  Data analysis strategy

Structural equation modelling (SEM) is integrated into the proposed model for data analysis in this study, for two main reasons. First, SEM allows the modelling of latent variables (e.g. perceived usefulness and perceived ease of use) that cannot be directly observed. Elderly people’s acceptance and demand for ICT products often involves subjective feelings and psychological factors, which are difficult to be directly measured by a single indicator, and SEM can more accurately reflect the influence of these latent variables through a multi-indicator measurement model. Second, this study acquires a large amount of experience data on elderly people’s use of ICT products based on data mining techniques. SEM is able to effectively integrate these multi-source data and reveal the causal relationships between variables through path analysis, providing data support for the optimisation of design elements. This study uses SEM to empirically test the constructed TAM and its hypotheses. The measurement and structural models were analyzed using the software Amos 28.0. The model parameters, including the factor loadings, correlations between latent variables and the standardized path coefficients, were estimated using the Maximum Likelihood method.

The analysis consisted of the following steps: first, the reliability and validity of the measurement model was assessed using confirmatory factor analysis (CFA), with Cronbach’s alpha values above 0.7 indicating high reliability. Second, convergent validity requires factor loadings of more than 0.70 for each item, and composite reliability (CR) and average variance extracted (AVE) values of more than 0.70 and 0.50, respectively^45^. Discriminant validity requires verifying that the square root of the AVE value is greater than the correlation between the constructs of interest. Finally, scale validity was assessed by exploratory factor analysis (EFA), preceded by KMO measurements and Bartlett’s test using SPSS 26.0, with KMO values above 0.6 and *p* < 0.05 indicating suitability for EFA analysis.

## Results

Our sample consisted of 174 elderly people (78 males, 96 females) aged 60 years and older. The demographic details of the respondents are presented in Table 5. During offline surveys, it was found that the first smartwatch of the elderly is commonly obtained as gifts from younger family members. This pattern accounts for the low adoption of smart products among the elderly and aligns with observations from online reviews. Many comments in these reviews are often written from the perspective of younger family members and describe the elderly’s experience with using smartwatches. For the question *“What factors do you believe may hinder your use of smartwatches?”*, the results show that 104 elderly users, accounting for 72%, selected *“Complex operation and difficulty in use”* as a hindrance. The proportion of users selecting *“Poor user interface experience*,*” “Price factors*,*” “Too many features*,* not practical*,*”* and other factors decreases successively.

**Table 5:** Demographic characteristics of the respondents.

Profile	Sample composition	*N*
Gender	Male Female	78 96
Age	60–74 > = 75	119 55
Education level	Primary school Middle school High school Junior college Bachelor’s degree or higher	70 35 32 21 16
Living condition	Live alone Live with partner Live with children	37 77 60
Personal income per month	< = CNY 2999 CNY 3000–4999 CNY 5000 ~ 7999 > = CNY 8000	76 53 27 18

After importing the questionnaire data into SPSS 26.0, Cronbach’s alpha values for the nine variables ranged from 0.786 to 0.905, with an overall reliability coefficient of 0.939, indicating strong reliability. Amos software results showed that all item factor loadings exceeded 0.70, with CR values between 0.8 and 0.903, above the 0.7 threshold, and AVE values greater than 0.50, confirming convergent validity. Table 6 presents the factor loadings, Cronbach’s alpha, CR, and AVE values.

**Table 6:** Descriptive analysis and assessment of the measurement model.

Construct	Items	Factor loadings	Cronbachs’ ɑ	CR	AVE
Perceived usefulness (PU)	PU1 PU2 PU3	0.888 0.83 0.89	0.901	0.903	0.757
Perceived ease of use (PEOU)	PEOU1 PEOU2	0.891 0.737	0.793	0.800	0.669
Perceived enjoyment (PE)	PE1 PE2	0.941 0.884	0.905	0.909	0.833
Behavioral intention (BI)	BI1 BI2 BI3	0.878 0.879 0.802	0.89	0.890	0.729
Functional architecture (FA)	FA1 FA2 FA3	0.846 0.866 0.786	0.871	0.872	0.694
Morphological aesthetics (MA)	MA1 MA2 MA3	0.708 0.826 0.733	0.786	0.801	0.574
Interaction mode (IM)	IM1 IM2	0.845 0.935	0.882	0.885	0.794
Human-computer interface (HCI)	HCI1 HCI2	0.916 0.898	0.9	0.903	0.823

Discriminant validity scores in Table 7 show diagonal values larger than the correlations below, confirming validity. The KMO measure was 0.883, exceeding the 0.6 threshold, and Bartlett’s test yielded a p-value of 0.000, confirming significance. Thus, the questionnaire is suitable for factor analysis. Principal component analysis (PCA) extracted 8 principal components, which accounted for a cumulative variance of 87.309%. This result exceeds the 70% threshold, signifying an effective summarisation of the 20 items (see Supplementary Table 2). In conclusion, the scale demonstrated good structural validity and accurately measured the intended concepts.

**Table 7:** Discriminant validity scores.

HCI	HCI	IM	FA	MA	PU	PEOU	PE	BI
	0.907							
IM	0.817	0.891						
FA	0.773	0.723	0.833					
MA	0.564	0.736	0.668	0.757				
PU	0.409	0.409	0.622	0.581	0.870			
PEOU	0.339	0.285	0.392	0.537	0.361	0.818		
PE	0.224	0.346	0.406	0.580	0.774	0.391	0.913	
BI	0.350	0.397	0.553	0.604	0.827	0.415	0.883	0.854

**Fig. 6:**
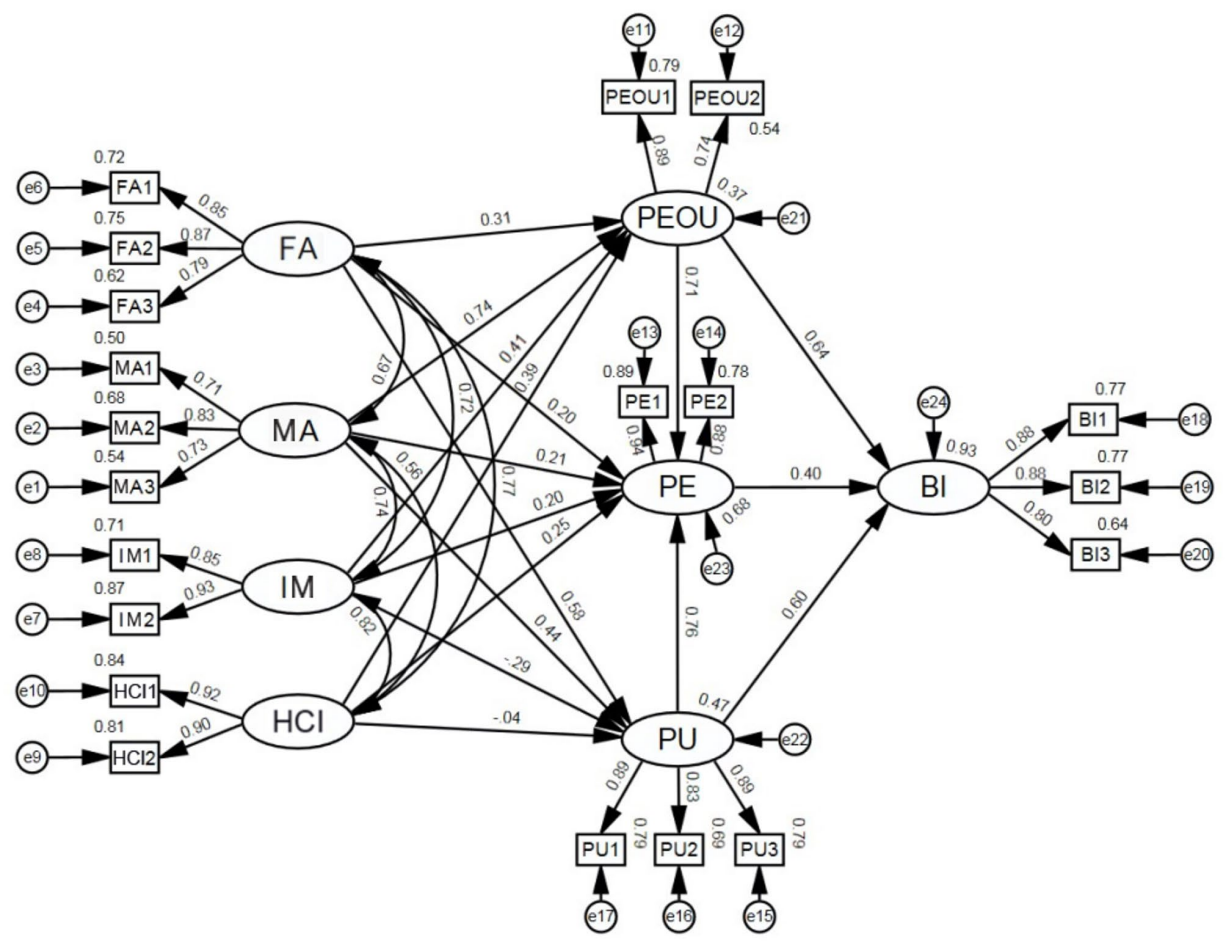
Structural equation model in Amos 28.0.

The corresponding structural equation model was constructed in Amos 28.0 software, as shown in Fig. 6. Various fit indices were calculated after running the model. The fit results indicate: CMIN/DF is 2.008, which is less than 3 and significant at *p* = 0.000; GFI = 0.891, AGFI = 0.801, IFI = 0.915, TLI = 0.887, CFI = 0.912, all greater than 0.8; RMSEA = 0.098, less than 0.1, indicating good fit. Thus, the model’s structural validity is adequate for further analysis based on the CFA results.

**Fig. 7:**
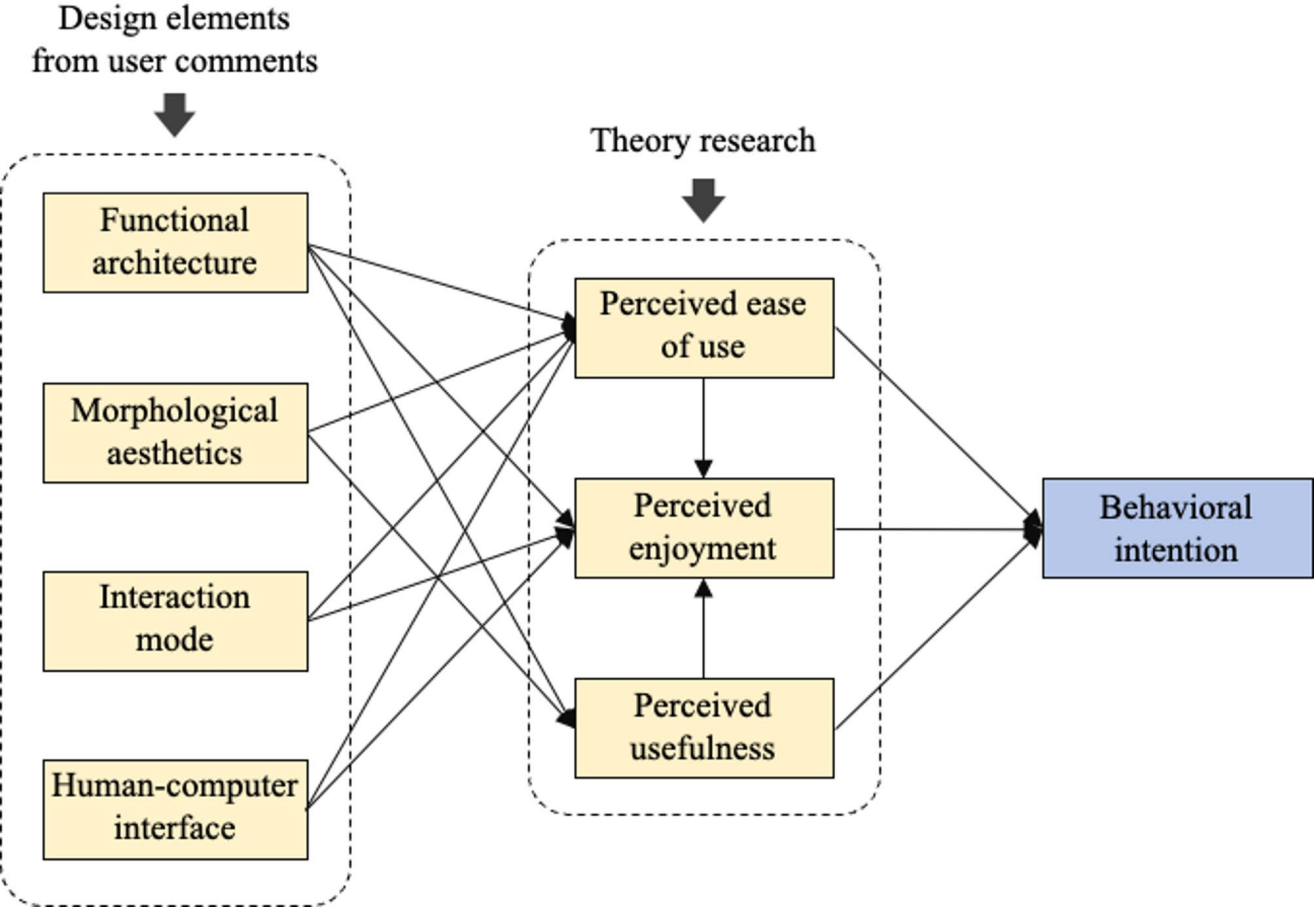
The UN-TAM proposed in this study.

Based on the proposed data mining model and modelling approaches described in “Model development and hypotheses” and “Results”, path analysis of the structural equation model was conducted using the Maximum Likelihood method in Amos 28.0. The results of the path analysis are summarised in Table 8. The standardized path coefficients reflect the magnitude of the relationships between variables, while *p*-values indicate significance. The *γ* coefficients indicate the extent to which the independent variables contribute to the explained variance of the dependent variables. The results indicate that out of 17 research hypotheses, 14 are supported, while 3 are not supported. The final UN-TAM, with non-significant correlations removed, is shown in Fig. 7.

**Table 8:** The results of path analysis and hypothesis test for the SEM.

Research hypothesis	Variable relationship	Standardized path coefficient (γ)	Significance	Hypothesis test
H1a	FA→PEOU	0.310	0.003**	Support
H1b	FA→PE	0.197	0.037*	Support
H1c	FA→PU	0.575	0.004**	Support
H2a	MA→PEOU	0.763	0.001**	Support
H2b	MA→PE	0.207	0.370	Nonsupport
H2c	MA→PU	0.435	0.022*	Support
H3a	IM→PEOU	0.414	0.042*	Support
H3b	IM→PE	0.255	0.030*	Support
H3c	IM→PU	-0.396	0.264	Nonsupport
H4a	HCI→PEOU	0.386	0.028*	Support
H4b	HCI→PE	0.251	0.003**	Support
H4c	HCI→PU	-0.042	0.858	Nonsupport
H5	PEOU→PE	0.712	***	Support
H6	PU→PE	0.757	***	Support
H7	PE→BI	0.402	***	Support
H8	PEOU→BI	0.641	***	Support
H9	PU→BI	0.601	***	Support

Statistical significance: *, p$$\:\le\:$$0.05; **, p$$\:\le\:$$0.01; ***, p$$\:\le\:$$0.001.

From the perspective of external variables in the model, and based on the standardized path coefficients, the following observations were made: product functional architecture significantly and positively affects perceived ease of use (*p* = 0.003), perceived usefulness (*p* = 0.004), and perceived enjoyment (*p* = 0.037), with the smallest effect on perceived enjoyment (*γ* = 0.197) and the greatest effect on perceived usefulness (*γ* = 0.575), supporting hypotheses H1a, H1b, and H1c; morphological aesthetics significantly and positively influences perceived usefulness (*p* = 0.022) and perceived ease of use (*p* = 0.001), with a smaller impact on perceived usefulness (*γ* = 0.435) and a greater impact on perceived ease of use (*γ* = 0.763), supporting hypotheses H2a and H2c; interactive mode significantly and positively affects perceived ease of use (*p* = 0.042) and perceived enjoyment (*p* = 0.030), with a greater impact on perceived ease of use (*γ* = 0.414) and a smaller impact on perceived enjoyment (*γ* = 0.255), supporting hypotheses H3a and H3b; human-computer interface significantly and positively influences perceived ease of use (*p* = 0.028) and perceived enjoyment (*p* = 0.003), with a greater impact on perceived ease of use (*γ* = 0.386) and a smaller impact on perceived enjoyment (*γ* = 0.251), supporting hypotheses H4a and H4b. The impact of morphological aesthetics on perceived enjoyment, and the impact of interaction mode and human-computer interface on perceived usefulness, are not significant; thus, hypotheses H2b, H3c, and H4c are not supported.

Overall, from the perspective of external variables, all four design elements significantly influence perceived ease of use. In addition, design elements significantly affecting the perceived enjoyment among the elderly include “functional architecture,” “interaction mode,” and “human-computer interface.” Design elements significantly influencing perceived usefulness among the elderly comprise “functional architecture” and “morphological aesthetics”.

In terms of the model’s internal variables, both perceived ease of use and perceived usefulness significantly and positively affect perceived enjoyment at the *p* less than 0.001 level; perceived ease of use perceived enjoyment and perceived usefulness significantly and positively affect behavioral intention at the same level. Therefore, hypotheses H5, H6, H7, H8, and H9 are all valid. Meanwhile, according to the magnitude of the standardized path coefficients, perceived ease of use has the greatest positive effect on behavioral intention (γ = 0.641), while perceived enjoyment has a relatively weak effect on behavioral intention (γ = 0.402). Perceived usefulness has the greatest positive effect on perceived enjoyment (γ = 0.757), surpassing the impact of perceived ease of use (γ = 0.712).

## Discussion

This study proposes a technology acceptance model integrating user needs, as shown in Fig. 4, which establishes a quantitative mapping framework between ICT product design parameters and elderly users’ behavioral intentions. Using smartwatches as a case study, the model demonstrates key design elements exhibit statistically significant effects on elderly users’ perceptual constructs.

### Key findings

#### Internal factors

Consistent with previous research conclusions, the model proposed in this paper once again confirms that perceived usefulness and ease of use have a direct and significant impact on the acceptance of smart wearable devices by the elderly^18,38,46^. In particular, perceived ease of use is the variable with the greatest overall effect in explaining technology acceptance, which is completely consistent with the previous research results^38^. Studies show that higher usability perceptions can mitigate fears related to new technologies, reflecting the elderly’s concerns about cognitive declines^18,38^. This aspect is also evident in the process of offline questionnaire collection. Common reasons for elderly people refusing to fill out questionnaires include statements like *“Never used it*,* don’t know how to use”* or *“Too complicated*,* can’t use it properly.”* These responses reflect the stereotype among the elderly that smart product operation is complex and difficult to handle, leading to resistance towards smart products. Thus, it is evident that perceived ease of use plays a decisive role in the acceptance of smartwatches by the elderly. Meanwhile, the model results also verified that product design elements can profoundly influence the perceived ease of use among the elderly, which is consistent with the conclusions of previous studies related to healthcare products^47^. To address psychological barriers hindering ICT adoption among the elderly, design improvements are necessary.

The elderly’s perceived usefulness significantly impacts their perceived enjoyment and behavioral intention, which also confirms the previous research results^18,38,46^. This analysis results can be explained by the elderly’s rational and utilitarian consumption beliefs and their relatively lower income levels. On the one hand, elderly individuals tend to exhibit mature and rational consumer traits, focusing on long-term benefits and maximizing utility when making purchasing decisions. For the elderly people with limited income, a product being deemed “useful’’ serves as the core driver for purchase. It must deliver sufficiently significant and irreplaceable value. In this context, the functional characteristics of ICT products become particularly crucial. The functional features of smartwatches are seen as tools to help with cognitive diseases and improve quality of life^18^. Smart wearable systems offer opportunities to address the issues related to aging, such as encouraging physical activity, monitoring vital signs, preventing isolation and contacting urgent care^48^. Research by Cota et al. also shows that the key factor for the positive impact of digital games on elderly individuals is their belief that these games are helpful in addressing cognitive health and enhancing quality of life^1^. Their emphasis on product utility is also evident in online reviews, where *“functional architecture”* and *“utility”* are frequently mentioned (Table 2). Therefore, particular attention should be paid to product functional design, which is crucial for promoting the acceptance of smartwatches among the elderly.

Unlike previous studies, the perceived enjoyment construct not only exerts a direct influence but also mediates the relationship between perceived usefulness and perceived ease of use on behavioral intention. In prior work, Ramírez-Correa et al. employed perceived enjoyment as a mediating variable between perceived ease of use and technology adoption among older adults, thereby enhancing model explanatory power^38^. Building upon this, the present study positions perceived enjoyment as a joint mediator for both perceived usefulness and perceived ease of use, with findings that corroborate this approach. This indicates that recognising a product’s usefulness and ease of use can promote positive emotional experiences among the elderly, thereby reducing the distance between users and products, as indicated in the literature^49,50^. Meanwhile, perceived enjoyment has a direct and significant impact on behavioral intention, which also supports previous studies^38,39,51^. In summary, in the design of ICT products, guiding positive emotions among the elderly ang enhancing their perceived enjoyment can effectively increase their willingness to use the product.

#### External factors

The model proposed in this study exhibits a notable divergence from existing research in the construction of external variables. The findings reveal that functional architecture influences older adults’ intrinsic perceptions through all three dimensions, which differs from the results of previous research due to differing emphases. For instance, Ma et al. demonstrated that specific functional features, such as health data monitoring, positively affect seniors’ perceived value and willingness to adopt^52^. In contrast, the present study explores the systemic impact of overall functional architecture design on users’ intrinsic perceptions. This conclusion is further substantiated by the data mining results from the first phase. As shown in Table 3, vocabulary related to functional architecture accounts for the largest proportion of all user requirements. Therefore, in the subsequent design process, particular attention should be paid to the design of the functional architecture. The design should be guided by three core principles: usability, ease of use, and user enjoyment.

Morphological aesthetics exerts a significant influence on both perceived ease of use and perceived usefulness, while the impact of morphological aesthetics on perceived enjoyment is not significant in this study, which contrasts with previous research findings^39,53^. This outcome also partially conflicts with the results of the earlier text mining analysis. There are a total of 635 vocabulary terms related to product appearance in online reviews, accounting for a considerable proportion of all review vocabulary. The discrepancy may be related to the design of the questionnaire items. The average score for the first question about morphological aesthetics, *“The appearance design of smartwatches is very important to me*,*”* was 3.2 points, the lowest average score among all items on the scale. This suggests that the elderly place a certain degree of importance on morphological aesthetics, but compared to other design elements, its priority is lower and does not reach the level of being “very important” among the options. Therefore, the design of morphological aesthetics should emphasize clarity in cognition and operation, while conveying a sense of reliability and professionalism. Specific design details can be further developed with reference to the findings from the user needs analysis based on text mining.

The interaction mode exerts a positive influence on users’ perceived ease of use, aligning with findings from existing studies on smart wearable devices for the elderly^54^. Moreover, this research further confirms that interaction mode also enhances perceived enjoyment, thereby extending the understanding of its affective mechanism. Wang et al. proposed that emotional design should be integrated into wearable health devices to enhance user enjoyment^55^. Hence, a successful interaction design should embody a synthesis of rationality and sensibility. It not only accomplishes tasks efficiently but also imparts a sense of achievement, control, and enjoyment to the elderly during use, thereby stimulating their usage intention on both cognitive and emotional levels.

The human–computer interface structure influences behavioral intention indirectly through its effects on perceived ease of use and perceived enjoyment. This finding both aligns with and diverges from prior research. On one hand, it partially corroborates previous conclusions. For instance, El-Gayar et al. characterized interface design through the concept of “readability” and verified its indirect effect on older adults’ behavioral intentions via mediating variables, consistent with the mechanism identified in this study^53^. On the other hand, Liu et al. found that interface element design exerts a direct rather than indirect influence on usage behavior^56^. Such differences may stem from variations in research contexts or model configurations. Despite these distinctions in influence pathways, both the present and previous studies underscore the pivotal role of interface design in the acceptance of intelligent technologies. Accordingly, this study suggests that the interface design of smart wearable devices for the elderly should follow a dual-path strategy: first, strengthen users’ confidence in use by reducing cognitive load; and second, stimulate interest in use by integrating emotional elements and pleasurable experiences.

### Contributions and limitations

The methodological innovation of this study is threefold. First, a participatory model development approach was proposed. It integrates online product reviews from elderly users through advanced data mining techniques. By embedding user feedback into the model development process, the user engagement is enhanced with an objective approach. Design parameters are derived from empirical behavioral data, as opposed to relying solely on hypothetical constructs. Second, this study improved the text mining process for specific user groups. An innovative “data purification” step based on identity-related keywords is conducted prior to the standard LDA topic modeling. This step effectively filters out relevant comments from target users, and the purity and representativeness of the subsequent analytical data are enhanced. This approach provides an efficient pathway to capture the needs of specific populations, such as the elderly people, from the large-scale online data. It serves as a supplement to the existing user demand acquisition methodologies. Finally, this study extends TAM within the field of industrial design, thereby enriching its relevance for design research and practice. By deconstructing the product at a macro level into four specific design elements, the study quantifies their influence on intrinsic perceptions and behavioral intentions of the elderly. This not only validates the applicability of TAM framework in design field but also provides solid theoretical foundation and definite optimisation directions for advancing age-friendly design.

Based on the above discussion, it should be noted that the proposed data-mining model and methodology still have some limitations. Firstly, due to the time and geographical constraints, the sample was collected from several provinces, including Sichuan, Anhui, and Shandong. It remains to be verified whether the findings from these provinces differ from those observed nationwide. Furthermore, additional investigation is needed to determine if core ICT product needs vary among elderly users based on gender, education level, income level, living condition and age group. For instance, our survey revealed that a majority of elderly users received their first smartwatch as a gift from their children. This suggests that living conditions, such as living with children, may facilitate device acquisition and usage support, thereby enhancing perceived ease of use. Conversely, living alone may present greater barriers to adoption. Therefore, it is essential to conduct user segmentation studies in the future research. Furthermore, the questionnaire survey revealed that price considerations constitute one of the primary barriers to adoption among the elderly. Meanwhile, most elderly people have relatively low incomes. A dedicated investigation into the mechanism of cost-effectiveness perception is needed to fully understand the decision-making processes of elderly users.

It is also worth noting that, as an initial exploration of data mining-driven model construction, validating the reliability of the proposed methodological pathway is one of the main contributions of the research. This lays a solid foundation for the later research. Building on the validated objective model, future research can develop basic constructs or mediation mechanisms. These new elements should be more closely aligned with the data-driven design attributes and the behaviors of the elderly.

## Conclusions

The present study has proposed and validated an innovative data-driven design optimisation model aimed at enhancing the elderly acceptance of ICT products. The main conclusions drawn from this study can be summarised as follows:

1. The data-driven approach can be employed to expand the methodological framework for examining the needs of elderly users. Online user reviews can serve as a novel avenue for gathering insights into their requirements. Practice in this study has shown that utilising keywords pertinent to the elderly identity to conduct “data purification” can effectively filters commentary data relevant to this demographic.
2. TAM demonstrates significant applicability within the field of industrial design. By integrating specific design elements with the TAM framework, a quantitative mapping relationship has been established from design parameters to user perceptions. This enables the model to be transformed from behavioral theory into an effective tool for guiding design practice. This exploration validates its cross-disciplinary explanatory power.
3. The established UN-TAM has elucidated the mechanisms on how product design can influence the acceptance of ICT products among the elderly, which can clarify the priorities in design. The findings of the present study have clearly indicated that: (a) The perceived usefulness, ease of use, and enjoyment significantly influence the acceptance of the elderly, with perceived ease of use being the most critical factor affecting their behavioral intention. (b) Different product design elements will variably affect the internal perception of the elderly, thereby influencing their behavioral intentions. Functional architecture notably enhances both perceived usefulness and ease of use, while morphological aesthetics primarily improves perceived ease of use. Additionally, interaction mode and human-computer interface positively impact perceived ease of use and enjoyment.

## Supplementary Information

Below is the link to the electronic supplementary material.

Supplementary Material 1

## Data Availability

Data will be made available from the corresponding author (Prof. Xiaogang Yang) or the first author (Yu Cao) upon reasonable request.
