# Supplementary material for: An innovative data mining-driven optimisation modelling approach based on TAM for the design of elderly-centric ICT products
Source: Sci Rep. 2026 Jan 24;16:6131. doi: 10.1038/s41598-026-37657-x (PMC12901014; doi:10.1038/s41598-026-37657-x)
Supplement: Supplementary file 1 — Supplementary Material 1 [file 41598_2026_37657_MOESM1_ESM.pdf]

Supplementary Material

Supplementary Table 1. The measurement variables and references of item settings.

Variable	Items	Measurement items	Sources or references
Perceived usefulness	a. PU1<br>b. PU2<br>c. PU3	a. Using smart watch can make my life more convenient.<br>b. Using a smartwatch gives me a better understanding of my physical condition.<br>c. Smart watch is very useful for me.	Davis, 19891/21/26 1:46:00 PM<br>Ren et al., 2022<br>Arning and Ziefle, 2007
Perceived ease of use	a. PEOU1<br>b. PEOU2	a. Smartwatches make me feel easy to use.<br>b. Smart watches make me feel easy to learn to use.	Davis, 1989<br>He and Huang, 2020
Perceived enjoyment	a. PE1<br>b. PE2	a. Using smart watch will make me feel happy.<br>b. Using a smart watch will make me feel the fun of technology.	Zhou and Zhou, 2021
Behavioral intention	a. BI1<br>b. BI2<br>c. BI3	a. Using a smartwatch is a very clear choice.<br>b. I would love to use a smartwatch.<br>c. Using a smart watch makes me feel meaningful.	Zhou and Zhou, 2021<br>Davis, 1989
Functional architecture	a. FA1<br>b. FA2<br>c. FA3	a. The function of a smartwatch is very important to me.<br>b. The streamlined function set will make me more willing to use a smartwatch.<br>c. Health monitoring, one-click call and voice call functions will make me more willing to use a smartwatch.	Ren et al., 2022
Morphological aesthetics	a. MA1<br>b. MA2<br>c. MA3	a. The appearance design of a smartwatch is very important to me.<br>b. I would prefer to use a smart watch in a reasonable color and size.<br>c. I would prefer to wear a smart watch that looks comfortable.	Yi et al., 2023<br>Huang et al., 2016
Interaction mode	a. IM1<br>b. IM2	a. The simple process is very important to me.<br>b. I would prefer to use a smart watch that operates naturally and smoothly.	\
Human-computer interface	a. HCI1<br>b. HCI2	a. I would be more willing to use a smart watch with less visual burden.<br>b. I would prefer to use a smart watch with a clear and simple interface.	\

Supplementary Table 2. Total variance explained.

C	IE			SSLE			SSLR		
	T	PV	CP	T	PV	CP	T	PV	CP
1	9.354	46.772	46.772	9.354	46.772	46.772	5.812	29.059	29.059
2	2.981	14.907	61.679	2.981	14.907	61.679	3.688	18.438	47.497
3	1.601	8.004	69.684	1.601	8.004	69.684	2.195	10.976	58.472
4	1.004	5.018	74.702	1.004	5.018	74.702	1.817	9.087	67.560
5	0.753	3.766	78.468	0.753	3.766	78.468	1.590	7.950	75.510
6	0.741	3.703	82.171	0.741	3.703	82.171	1.174	5.870	81.380
7	0.549	2.743	84.914	0.549	2.743	84.914	0.636	3.182	84.562
8	0.479	2.395	87.309	0.479	2.395	87.309	0.549	2.747	87.309
9	0.420	2.100	89.409						

10	0.343	1.716	91.125
11	0.272	1.360	92.485
12	0.250	1.250	93.735
13	0.220	1.102	94.837
14	0.206	1.028	95.865
15	0.193	0.964	96.829
16	0.167	0.836	97.665
17	0.144	0.720	98.386
18	0.131	0.655	99.041
19	0.102	0.511	99.552
20	0.090	0.448	100.000

Note: **C**, component; **IE**, initial eigenvalues; **SSLE**, sum of squares of loadings after extraction; **SSLR**, sum of squares of loadings after rotation; **T**, total; **PV**, percentage of variance; **CP**, cumulative percentage.
